# The Specificity of Metacognition Questionnaire-30 Subdimensions: Findings From Connectome-Based Predictive Modeling

**DOI:** 10.1155/da/5581270

**Published:** 2025-08-19

**Authors:** Ruocen Hu, Meng Yu, Liangfang Li, Hui He, Sihan Wei, Junji Ma, Yue Gu, Zhengjia Dai

**Affiliations:** ^1^Department of Psychology, Sun Yat-sen University, Guangzhou, Guangdong, China; ^2^Department of Psychology, School of Public Health, Southern Medical University, Guangzhou, Guangdong, China

**Keywords:** emotional distresses, functional connectivity, metacognition, resting-state functional magnetic resonance imaging

## Abstract

**Background:** The maladaptive metacognition measured by the Metacognition Questionnaire-30 (MCQ-30) is often linked to a wide range of affective disorders. However, few studies have elucidated the neural underpinnings of different metacognition subdimensions. Additionally, the relationship between these functional neural bases and longitudinal changes in individual emotional distresses remains unclear.

**Methods:** A total of 180 college students completed brain imaging and a battery of behavioral assessments. Employing the connectome-based predictive modeling (CPM), we delineated the functional connectivity (FC) network of each metacognition subdimension. Then, the mediation model was used to explore the relationships between FC networks, metacognition subdimensions, and emotional distresses.

**Results:** Default mode network (DMN) was found to be the general network of three significant subdimensions. Specifically, the FC network of cognitive self-consciousness (CSC) was scattered and mainly relied on DMN and frontoparietal network; need to control thoughts (NC) was largely consisted of the correlates between DMN and ventral attention network (VAN); negative beliefs about uncontrollability and danger of worry (NEG) was primarily associated with DMN and its correlates with visual network. CSC, NC, and NEG could mediate the relationship between the corresponding FC network and emotional distresses. Additionally, the CSC related and NEG related FCs could effectively predict the change of anxiety positive affect (PA) and negative affect (NA).

**Conclusions:** These findings demonstrated the common and distinct FC bases of maladaptive metacognition. The excessive FCs of CSC and NEG might be responsible for impaired self-check-related ability and further increase the risk of several affective disorders.

## 1. Introduction

Metacognition is the cognition about cognition [1], which refers to the knowledge and belief of the cognitive subject about the self-cognitive system, as well as the evaluation and adjustment strategies of self-cognitive activities [2]. The meta-cognitive model in psychopathology, proposed by Wells and Matthews [3], suggests that the dysfunction of metacognition could contribute to various psychological disorders. This model further links metacognition with the specific pattern of responding known as cognitive attention syndrome (CAS). The CAS is characterized by repetitive negative thinking (i.e., rumination and worry), threat-focused attention, and other maladaptive coping strategies that intensify and prolong emotional response [4, 5]. Meta-cognitive model particularly emphasizes the determining role of metacognition and CAS in triggering anxiety and depression. That is, CAS caused by maladaptive metacognition would guide individual towards a particularly toxic style of sustained and inflexible conscious processing of negative thoughts and feelings, further leading to the maintenance and development of psychological disorders [6–8]. Hence, metacognition is an essential factor for mental health outcomes in both healthy populations and clinical samples [5, 9–12].

The metacognition widely measured by the Metacognition Questionnaire (MCQ)-30 [13], which focuses on how individuals respond to those maladaptive thoughts, consists of five stable subdimensions. Specifically, positive beliefs about worry (POS) and negative beliefs about uncontrollability and danger of worry (NEG) evaluate the positive and negative beliefs related to maladaptive metacognition. The cognitive self-consciousness (CSC) dimension focuses on the metacognitive monitoring process of self-awareness, while the need to control thoughts (NC) dimension reflects individuals' awareness of the need to control thoughts, and Cognitive Confidence (CC) measures judgments of CC. The associations observed between these metacognition subdimensions and various psychological disorders suggest both shared characteristics and distinctive features, indicating divergent metacognitive profiles among different disorders [5].

Related convergent evidence indicated that the NEG dimension is prominent in individual anxiety [14] and is the characteristic factor to predict the development of both anxiety and depression [15]. In a systematic review by Capobianco et al. [6], NEG significantly and positively predicted symptoms of anxiety and depression across physical illnesses, while POS showed less consistency and weaker associations with anxiety and depression. A recent empirical study also found that NEG is the subdimension most strongly correlated with anxiety and depression, followed by CSC and NC. Among these, CC showed the lowest correlation, while POS demonstrated a stronger correlation with anxiety rather than depression (Ådnøy et al. [16]). Nevertheless, Aldahadha [9] revealed a significant effect of NEG, CC, and NC on depression level. However, these effects were not observed across all metacognition subdimensions and anxious levels. Hence, each subdimension of metacognition plays a distinct role in the maintenance and development of affective disorders, which are probably due to differences in the underlying neural basis of the maladaptive metacognition subdimensions.

The progress in neuroimaging studies offers new insights to inform our understanding of the neural bases of metacognition. However, fMRI studies investigating metacognition through a mental health lens remain scarce, particularly using the MCQ-30 as a measurement tool. In contrast, studies on the neural bases of metacognition related to cognitive performance or abilities have accumulated more results. For instance, Seow et al. [17] suggested that the ventromedial prefrontal cortex (PFC) reflects self-performance estimates, while the frontopolar cortex and the lateral PFC serve as central hubs in mediating explicit metacognitive judgments and (meta)cognitive control. Some other nodes linked to metacognitive evaluation exhibit cognitive domain-specificity, for example, the precuneus has primarily been implicated in memory metacognition [18], while lateral–parietal areas are related to individual metacognitive perception [19]. Of note, most of these regions are known to be the key parts of the default mode network (DMN) [20, 21], thus, it is reasonable to highlight the importance of DMN for metacognition, although its specific substructures might be related to different metacognition aspects. In studies of CAS related to maladaptive metacognition, research has demonstrated that core features such as rumination and worry, are involved in multiple interconnected brain networks. Specifically, these features are associated with the DMN and critically engage other key networks, including the affective, salience, and executive networks [22–24]. Kowalski et al. [25] provided further insights by revealing that high levels of CAS correlate with significant disruptions in connectivity patterns—both within and between the DMN, salience, and central executive networks. These findings suggest the neural bases of metacognition as fundamentally distributed and interconnected systems. Resting-state functional connectivity (FC), which measures temporal correlations between spatially distinct brain regions using resting-state fMRI (R-fMRI) data [26], has emerged as a powerful analytical approach. Researchers have utilized large-scale FC data to map psychological processes to comprehensive brain networks, enabling a deeper understanding of complex cognitive functions [27–31]. Resting-state FC has been shown to be associated with various cognitive domains [32] and can serve as a reliable predictor of individual differences in cognitive functioning [33], which suggests its potential value as a neural marker for investigating individual variations in metacognition ability. Furthermore, FC has also been employed to explore the neural mechanisms of metacognition [34–38]. For instance, to the best of our knowledge, there is currently only one brain imaging study focusing on the MCQ-30, which found that the total score of the MCQ-30 was associated with the FC of three regions of interest (ROIs): the right superior temporal gyrus, fusiform gyrus, and left anterior central gyrus [35]. However, comparisons of the neural bases of different metacognition subdimensions from a whole-brain network perspective are still lacking.

Using the whole-brain–based FC network analysis and connectome-based predictive modeling (CPM), the current study aims to identify: 1) the neural basis of the metacognition subdimensions measured by MCQ-30 and 2) the relationship between the neural basis of metacognition subdimensions, the scores of each subdimension and the emotional distresses (i.e., anxiety and depression, as well as more universal emotional states: positive affect (PA) and negative affect (NA)). Furthermore, based on the longitudinal prediction, this study focuses on whether the FC of a specific subdimension of metacognition can be an effective predictor for the changes in emotional distress scores. It is worth mentioning that the specificity discussed here does not imply a clear directional assumption, but rather an exploratory association. CPM is a recent approach to predict individual behavior or symptoms in psychiatric disorders from whole-brain FC data [39–41] and has demonstrated stable test–retest reliability in several studies involving longitudinal data [42–44]. Compared to the traditional ROI approach, the data-driven CPM does not require preselected ROIs, and is effective to facilitate the identification of large-scale networks related to metacognition.

## 2. Materials and Methods

### 2.1. Participants

A total of 181 undergraduates were recruited through online/printed posters from the Guangzhou Higher Education Mega Center and completed the T1-weighted structural imaging, R-fMRI scanning, and online behavioral questionnaires. All participants were screened for no clinical anxiety or depression symptoms using the Beck Anxiety Inventory (BAI) and Beck Depression Inventory-II (BDI-II). Other inclusion criteria included: (1) right-handed Han Chinese, (2) not currently receiving any psychotherapy or prescription medication, (3) having no suicidal tendencies, and (4) having no history of neurological or psychiatric disorders. One subject was excluded due to excessive head motion (>2 mm or 2°) during the scan, resulting in a final 180 subjects (103 females; 19.09 ± 0.84 years old; age range 18–21 years). All participants had given informed consent and this study was approved by the Institutional Review Board in the Department of Psychology at Sun Yat-sen University (ethics approval number: 2019-1024-0107).

Among 180 participants in the initial test (t1), repeated behavioral assessments were conducted in 159 of them (96 females; 20.18 ± 0.82 years old; age range 19–22 years) after 12–18 months (t2) to investigate the longitudinal changes of emotional distress variables. To control the effect of different measurement intervals intersubjects, both retest scores and the change scores of emotional distress variables (t1 vs. t2) were time-processed ((score/measurement interval) × 365).

### 2.2. Image Acquisition and Data Preprocessing

The MRI images were collected using a 3T Siemens Prisma scanner (Siemens, Germany). T1-weighted structural images were obtained with the following parameters: repetition time (TR) = 2500 ms, echo time (TE) = 2.38 ms, flip angle (FA) = 8°, field of view (FOV) = 320 × 320 mm^2^, data matrix = 320 × 320, and voxel size = 0.8 × 0.8 × 0.8 mm^3^. The R-fMRI images were acquired using a gradient echo plane with the following parameters: TR = 2000 ms, TE = 30 ms, slices = 37, slice thickness = 3.5 mm, FA = 90°, volumes = 240, FOV = 224 × 224 mm^2^, data matrix = 64 × 64, and voxel size = 3.5 × 3.5 × 3.5 mm^3^. The resting-state fMRI data were acquired over a duration of 8 min, during which 240 volumes (TRs) were collected. Participants were asked to close their eyes, stay still and awake, without thinking anything else during the scanning.

Imaging data were preprocessed using data processing assistant for resting-state fMRI toolbox (http://rfmri.org/DPARSF, V5.2_210501; Yan et al. [45]) with the following steps: (1) removing the first 10 volumes; (2) slice timing correction; (3) head motion correction; (4) registering the T1-weighted images to the functional image, and segmenting the transformed structural images into white matter, gray matter and cerebrospinal fluid using a unified segmentation algorithm; (5) transforming the head motion corrected functional images to the Montreal Neurological Institute (MNI) standard space using the normalization parameters estimated during unified segmentation, and the voxel size was then resampled to 3 × 3 × 3 mm^3^; (6) smoothing the functional images with Gaussian kernel function (full width at half maximum = 4 mm); (7) detrending and regressing out the nuisance signals including white matter signals, cerebrospinal fluid signals, global mean signals, and Friston-24 head motion parameters; (8) temporal filtering (bandpass, 0.01–0.08 Hz).

After data preprocessing, each brain image was divided into 264 ROIs using the Power264 template [46] and the mean blood oxygen level dependent (BOLD) signals of all the voxels in each ROI were extracted using the GRETNA toolbox v2.0.0 [47]. The 264 distinct ROIs were typically grouped into 14 functional networks: sensory/somatomotor hand network (SMH); sensory/somatomotor mouth network (SMM); cingulo-opercular task control network (CON); auditory network (AUD); DMN; memory retrieval network (MRN); visual network (VIS); frontoparietal task control network (FPN); salience network (SN); subcortical network (SUB); ventral attention network (VAN); cerebellar network (CN); dorsal attention network (DAN); uncertain network (UNC). Pearson's correlation coefficients between mean BOLD signals of each pair ROIs were calculated and then transformed to Fisher's *z*-scores as the FC edges. Thus, each subject obtained a symmetric 264 × 264 whole-brain FC matrix for further analyses.

### 2.3. Behavioral Assessment

Given the widespread use of the MCQ-30 globally [5, 48–50], the Chinese version of the MCQ-30 was utilized to assess maladaptive metacognition [11, 13]. It includes 30 items in five subdimensions referring to positive and negative beliefs of worry (i.e., POS and NEG), metacognitive monitor and control (i.e., CSC and NC), and judgement of CC. In the current study, Cronbach's *α* coefficients of total MCQ-30 were 0.860 in t1 and 0.874 in t2, while the five subdimension scores varied from moderate to high internal consistency across t1 and t2 (for more detailed information please see Table S1).

Positive and negative mood were assessed using the Chinese version of positive and negative affect schedule (PANAS) [51, 52]. The questionnaire includes two dimensions, with 8 items rating PA and eight items rating NA. In this study, Cronbach's *α* coefficients of PA were 0.891 in t1 and 0.885 in t2, NA were 0.763 and 0.863, respectively.

The 21-item BAI [53] and 21-item BDI-II [54, 55] were adopted to assess the degree of anxiety and depressive emotions, respectively. Cronbach's *α* coefficients of BAI in this study were 0.847 in t1 and 0.900 in t2, and those of BDI-II were 0.862 and 0.896, respectively.

### 2.4. Constructing CPM

Here, the CPM method [39] was used to establish the prediction model of brain and metacognition through the leave-one-out cross-validation (LOOCV) based on FC in t1. First, we examined the relationship between each edge and metacognitive measures (i.e., the total score of MCQ-30 and its five metacognition subdimensions) using correlation analyses, controlling for gender, age, and head motion as covariates. Following previous studies [39, 56–58], edges showing significant positive or negative correlation (*p* < 0.01) with metacognitive measures were selected as the features and categorized into two groups based on their correlation direction with behavioral measures: behaviorally positive-correlated and behaviorally negative-correlated networks. Next, within each network, we summed all significant FC edges, as these edges demonstrated consistent directional relationships with target behavior, regardless of their FC sign. By summing these FC values, we can quantify the cumulative FC contribution while preserving their unified behavioral associations, independent of the directionality (positive or negative) of the original FC. Then, for each network, linear regression was applied for the summed feature edge strength to build the prediction models. In each iteration, Pearson's correlations between the predicted metacognition scores and observed ones were calculated and significant positive *r* values were used as valid results. A permutation test (1000 times) was used to evaluate the statistical significance. Eventually, CPM provided a clear analysis to identify the predictive connectome with the most relevant FC features for each metacognition subdimension. We separately built predictive models based on the edges that positively and negatively correlate with metacognitive scores, resulting in six positive-correlated FC networks and six negative-correlated FC networks. More detailed information was provided in Supporting Information.

### 2.5. Statistical Analysis

The mean values and standard deviations of the MCQ-30, PA, NA, BAI, and BDI were calculated using SPSS 22.0. As for longitudinal changes between t1 and t2, the paired samples *t*-test was conducted. Pearson's correlation analyses were used to explore the relationships between metacognition and individual levels of anxiety, depression, PA, and NA in both t1 and t2. Model 4 of the PROCESS macro for SPSS [59] was used for mediation analyses to investigate the mediating effect of metacognition subdimension scores (mediating variables) in the relationship between FC (independent variable) and PANAS scores, BAI scores, and BDI scores (dependent variables, respectively). Here in mediation analyses, all the variables were measured in t1. Age, gender, and head motion were controlled as the covariates. The value of the independent variable is the mean value of the FC edges that can predict the score of the metacognition subdimension in the prediction model. The mediating effect is valid, if the bootstrapped (5000 times) 95% confidence interval of the coefficient does not contain 0. Moreover, to examine whether t1 metacognition subdimensions could mediate the relationships between t1 FC networks and t2 emotional outcomes, we conducted additional mediation analyses based on emotion outcomes (PANAS, BAI, and BDI scores) measured in t2 as dependent variables.

To better understand the impact of maladaptive metacognition on the long-term development of emotional distresses, we constructed the CPM model again based on the underlying FC networks, which were significantly related to metacognition subdimensions (i.e., CSC, NEG, and NC) in t1 and applied it to predict the score changes of emotional distresses. The change of emotional distresses was calculated as: ((t2 score - t1 score)/measurement interval; Supporting Information).

### 2.6. Validation Analysis

To ensure the robustness of our findings, we conducted comprehensive validation analyses. In the realm of CPM studies,the LOOCV procedure has been commonly utilized recently [60–62]. However, we recognized the potential of *K*-fold cross-validation in enhancing predictive model generalizability [63, 64]. For this reason, we opted to conduct additional analyses using tenfold cross-validation. We repeated the tenfold cross-validation 100 times, averaged the predicted values for each subject and assessed the relationship between averaged predicted values and empirical values using Pearson's correlation, with statistical significance evaluated through permutation testing (1000 iterations).

To mitigate the potential impact of motion-related confounds in FC estimation, we implemented a scrubbing procedure concurrent with nuisance signal regression (white matter signals, cerebrospinal fluid signals, global mean signals, and Friston-24 head motion parameters). Specifically, time points with framewise displacement (FD) exceeding 0.5 mm were identified as motion-contaminated and removed. The FC matrices were then reconstructed using the cleaned time series prior to CPM analysis.

Furthermore, we performed sensitivity analyses for the significant metacognition subdimensions across multiple edge-selecting *p* thresholds (*p* < 0.025, 0.01, 0.005, and 0.001), in order to test the rationality of threshold selection. On this basis, to more fully assess the reliability and template specificity of our findings, we conducted parallel analyses using two additional parcellation schemes: the Shen 268-node atlas [65] and the Brainnetome atlas (246 nodes) [66], maintaining the original edge-selection threshold (*p* < 0.01).

## 3. Results

### 3.1. The Correlations Between Metacognition and Emotional Distresses

The descriptive results showed that the scores of PA (*t* = 2.231, *p*=0.027) and BAI (*t* = 3.675, *p* < 0.001) were significantly decreased, while BDI was significantly increased (*t* = –7.015, *p*  < 0.001) between t2 and t1, more details can be found in Table S1. The correlation analyses showed significant positive relationships between the scores of MCQ-30 subdimensions and emotional distresses (i.e., BAI, BDI, PA, and NA) in t1, as shown in Table 1. In the t1, the CC and NC subdimensions were significantly positively correlated with BAI, BDI, and NA; the POS subdimension was significantly correlated with PA and BDI, while the CSC subdimension had significant positive correlations with NA and BAI, and a marginal positive correlation with PA; the NEG subdimension was significantly correlated with all emotional variables.

### 3.2. CPM Results: FC Network of Metacognition Subdimensions

#### 3.2.1. Predictive Network Selection

As shown in Table 2, the results of CPM prediction models and permutation tests (1000 times) indicated that three FC networks were found to significantly predict metacognition subdimensions. Specifically, the positive-correlated FC networks of CSC (*r* = 0.204, *p*_perm_ = 0.033, and *p*_fdr_ = 0.033) and NC (*r* = 0.238, *p*_perm_ = 0.015, and *p*_fdr_ = 0.030) were significant after FDR correction and the negative-correlated FC network of NEG (*r* = 0.212, *p*_perm_ = 0.023, and *p*_fdr_ = 0.092) was significant at the uncorrected level and marginally significant after FDR correction. These findings were further validated through 5000 iterations of permutation tests (Table S7). Given this consistency, along with our comprehensive validation analyses, and considering the substantially higher computational demands of 5000 permutations, we decided to use 1000 permutation tests in the main analysis and validation analysis.

#### 3.2.2. FC Network of CSC, NC, and NEG Subdimensions

**Figure 1:**
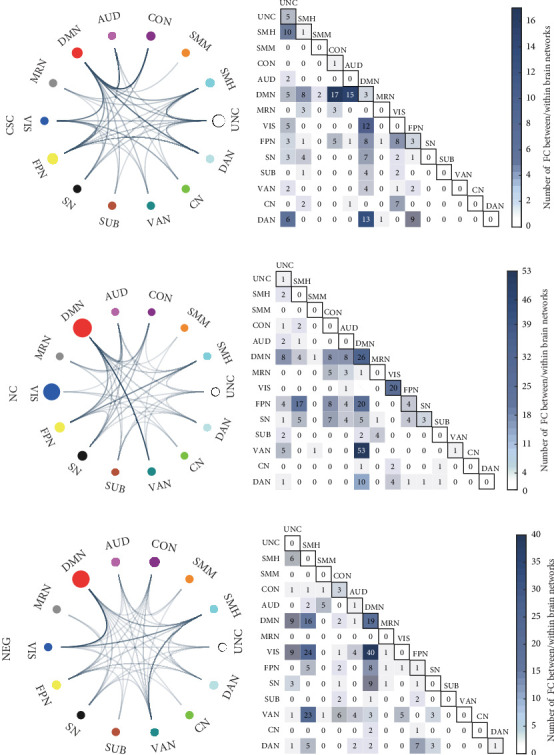
Visualization of the significant predictive network results from CPM models in predicting CSC, NC, and NEG subdimensions. (A) Chord plots of predictive edges in the positive-correlated FC network for CSC subdimension. The circles in the chord plot represent networks, which were color-coded according to 14 canonical networks by Power et al. [46]: AUD, auditory network; CN, cerebellar network; CON, cingulo-opercular task control network; DAN, dorsal attention network; DMN, default mode network; FPN, frontoparietal task control network; MRN, memory retrieval network; SMH, sensory/somatomotor hand network; SMM, sensory/somatomotor mouth network; SN, salience network; SUB, subcortical network; UNC, uncertain; VAN, ventral attention network; VIS, visual network. The lines connecting the circles represent functional connections between networks. The darkness of the lines reflects the number of connections, where a darker line indicates a denser set of internetwork connections. The size of the circles represents the connection strength within the network, with a larger circle indicating a higher number of intranetwork connections. (B) Heatmap showing the number of predictive edges consisted the positive-correlated FC network for CSC subdimension. The number in the diagonal square represents the number of intranetwork FCs within each network and the remaining square represents the number of internetwork FCs between two networks. The depth of the color bar indicates the quantity of significant FC. (C) Chord plots of predictive edges in the positive-correlated FC network for NC subdimension. (D) Heatmap showing the number of predictive edges consisted the positive-correlated FC network for NC subdimension. (E) Chord plots of predictive edges in the negative-correlated FC network for NEG subdimension. (F) Heatmap showing the number of predictive edges consisted the negative-correlated FC network for NEG subdimension.

The positive-correlated FC network of the CSC subdimension contains 194 edges, an overwhelming majority of which were inter-network edges (181 edges accounting for 93.30%), including the FCs between DMN and the CON (17 edges), DMN-AUD (15 edges), DMN–DAN (13 edges), and DMN–VIS (12 edges). The right precentral gyrus (BA6, MNI coordinates = 29, −5, 54) made the highest contribution in predicting, as well as the other two nodes with high degree (that is the nodes have more direct edges than other nodes): the left angular (BA39, MNI coordinates = −42, −55, 45) and the right cerebellum Crus1 (MNI coordinates = 35, −67, −34). Refer to Figure 1a,b and Table S2 for specific information.

The positive-correlated FC network of NC subdimension contains 271 edges, 216 (79.70%) of which were internetwork edges and 55 (20.30%) were intranetwork edges. The internetwork edges were mainly among the high-order functional models, including FCs between DMN–VAN (53 edges), DMN–FPN (20 edges), FPN–SMH (17 edges), and DMN–DAN (10 edges). Regarding the node, the degree of 38 made the right superior temporal gyrus (BA22, MNI coordinates = 52, −33, 8) with the highest contribution to the prediction model, followed by the right precentral gyrus (BA38, MNI coordinates = 29, −5, 54), and the right medial superior frontal gyrus (BA10, MNI coordinates = 9, 54, 3). Detailed information is given in Figure 1c,d and Table S3.

The negative-correlated FC network of NEG subdimension contains 254 edges, excluding 25 intranetwork edges (9.84%), most of which (229 edges, 90.16%) were internetwork FCs, mainly found between VIS and DMN (40 edges), VIS–SMH (24 edges), VAN–SMH (23 edges), and DMN–SMH (16 edges). The right superior temporal gyrus (BA22, MNI coordinates = 52, −33, 8) with the highest node degree (24 edges) showed greater contribution to the prediction model again, followed by the left middle temporal gyrus (BA21, MNI coordinates = −58, −30, −4), and the right supplementary motor area (BA6, MNI coordinates = 3, −17, 58), et cetera (Figure 1e,f and Table S4).

#### 3.2.3. Coincidence Analysis of FC Networks in Dimensions of Metacognition

**Figure 2:**
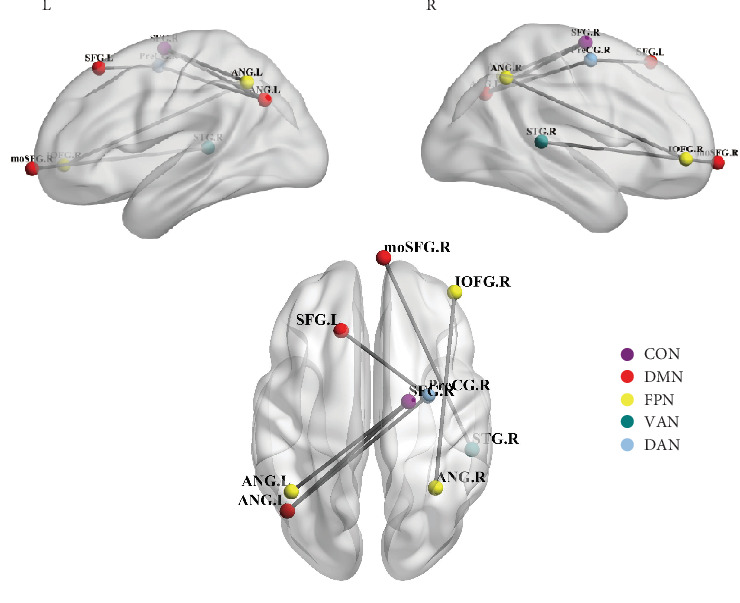
The common edges shared by predictive networks related to CSC and NC subdimensions. The overlapping edges were identified by coincidence analysis and were visualized using the BrainNet viewer toolbox (http://www.nitrc.org/projects/bnv/) [67]. In this glass brain, lines indicate the FC edges; nodes are labeled and colored according to the network they belong to. CON, cingulo-opercular task control network; DAN, dorsal attention network; DMN, default mode network; FPN, frontoparietal task control network; VAN, ventral attention network. Brain region abbreviations: SFG.L, left superior frontal gyrus; ANG.L, left angular gyrus; moSFG.R, right medial orbital superior frontal gyrus; IOFG.R, right inferior orbital frontal gyrus; PreCG.R, right precentral gyrus; SFG.R, right superior frontal gyrus; STG.R, right superior temporal gyrus; ANG.R, right angular gyrus.

To investigate the common and distinct patterns among the FC bases of CSC, NC, and NEG subdimensions, we conducted a coincidence analysis by intersecting the FC networks. Specifically, we performed pairwise intersections of the FC edges among the three dimensions—CSC, NC, and NEG—to investigate whether there are completely identical FC edges shared between any two dimensions of the FC networks. The FC networks that predict the three subdimensions of NEG, CSC, and NC do not share any overlapping edges among them. Similarly, there is no overlap between the NEG FC network and the FC networks of CSC or NC. However, six overlapping edges related to the high-level networks (e.g., DMN, DAN, VAN, FPN, and CON) were presented in both the positive-related FC network of CSC and NC, as illustrated in Figure 2 and Table S5.

### 3.3. Mediation Effect Analysis

Taken together, the results of assessing indirect effects showed that: (1) the effect of CSC FC network on BAI/NA was significantly mediated by CSC score (indirect effect was 0.166 and 0.182, respectively); (2) the effect of NC FC network on BAI/BDI/NA was mediated by the level of the NC (indirect effect was 0.366, 0.337, and 0.399, respectively); (3) the effect of NEG FC network on BAI/BDI/PA/NA was significantly mediated by the level of the NEG (indirect effect was −0.315, −0.366, 0.184, and −0.380, respectively). More detailed results are shown in Table 3. The mediation analyses based on t2 emotion outcomes did not yield significant mediation results (Table S6).

### 3.4. CPM Results: Longitudinal Prediction Analysis

**Figure 3:**
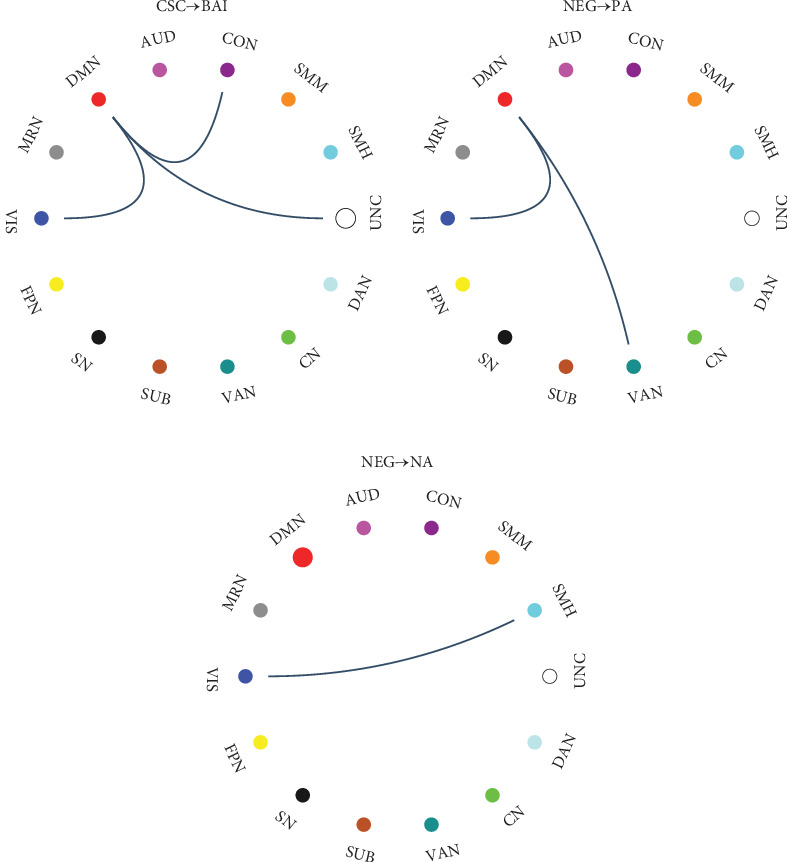
Chord plot for FC edges that can longitudinally predict emotional distresses. (A) CSC→BAI: the predictive FC edges of CSC subdimension that can predict BAI changes; (B) NEG→PA: the predictive FC edges of NEG subdimension that can predict PA changes; (C) NEG→NA: the predictive FC edges of NEG subdimension that can predict NA changes. The circles in the chord plot represent networks, which were color-coded according to 14 canonical networks: AUD, auditory network; CN, cerebellar network; CON, cingulo-opercular task control network; DAN, dorsal attention network; DMN, default mode network; FPN, frontoparietal task control network; MRN, memory retrieval network; SMH, sensory/somatomotor hand network; SMM, sensory/somatomotor mouth network; SN, salience network; SUB, subcortical network; UNC, uncertain; VAN, ventral attention network; VIS, visual network. The lines connecting the circles represent functional connections between networks. The darkness of the lines reflects the number of connections, where a darker line indicates a denser set of inter-network connections. The size of the circles represents the connection strength within the network, with a larger circle indicating a higher number of intranetwork connections.

Further, the CPM model result suggested that the FC networks of CSC and NEG in t1 could be the most significant predictors of score changes in individuals' emotional distresses between t1 and t2 (Figure 3). Amidst the functional connectome of CSC detected in t1, the internetwork FC edges of DMN-VIS/CON/UNC and within-network FC of UNC could predict the longitudinal change of BAI (*r* = 0.175, *p*=0.027). In comparison to CSC, the FC networks of NEG were more strongly related to the change of PANAS. The internetwork FC edges of DMN–VIS and DMN–VAN could marginally predict the changes of PA (*r* = 0.139, *p*=0.080), while the VIS–SMH internetwork FC and one FC edge within DMN significantly predicted changes of NA (*r* = 0.179, *p*=0.024). No significant prediction effect of the NC FC edges was found.

### 3.5. Validation Analysis

The CPM results based on the tenfold cross-validation largely converged with LOOCV results (Table S8). The positive-correlated FC network in the NC subdimension was significant at the uncorrected level and marginally significant after FDR correction (*r* = 0.191, *p*_perm_ = 0.024, and *p*_fdr_ = 0.096). Moreover, the negative-correlated FC network in the NEG subdimension was significant (*r* = 0.215, *p*_perm_ = 0.006, and *p*_fdr_ = 0.024). However, the significant prediction for the positive-correlated FC network in the CSC subdimension based on LOOCV was not replicated.

The results from the head-motion-scrubbing validation analysis are consistent with our main findings (Table S9). Specifically, the prediction result of the positive-correlated FC network for the CSC subdimension was significant (*r* = 0.206, *p*_perm_ = 0.024, and *p*_fdr_ *=* 0.024), as was the result of positive-correlated FC network for the NC subdimension (*r* = 0.228, *p*_perm_ = 0.013, and *p*_fdr_ *=* 0.024). Additionally, the negative-correlated FC network for the NEG subdimension was also significant at the uncorrected level (*r* = 0.209, *p*_perm_ = 0.027, and *p*_fdr_ *=* 0.108). These convergent results further supported the robustness of our findings, indicating that the identified neural bases were not driven by head motion artifacts.

Sensitivity analyses indicated that thresholds of *p* < 0.01 and *p* < 0.005 strike a reasonable balance between selecting enough FC features and preventing model overgeneralization (Tables S10, S11, and S12). At more stringent thresholds (*p* < 0.005 or 0.001), significant predictive effects for the NC and NEG subdimensions of the FC networks were found, potentially supporting the reliability of results at the *p* < 0.01 level.

Using the Shen 268-node atlas (Table S13), significant predictions were found for both the positive-correlated FC network of NC (*r* = 0.302, *p* < 0.001) and the negative-correlated FC network of NC (*r* = 0.193, *p*=0.009). With the Brainnetome 246-node atlas (Table S14), significant predictions emerged for the positive-correlated FC network of NC (*r* = 0.296, *p* < 0.001), as well as the negative-correlated FC network of NC (*r* = 0.230, *p*=0.002) and NEG (*r* = 0.165, *p*=0.027).

## 4. Discussion

In the current study, we combined the CPM and mediation model to explore the neural connections of each subdimension of maladaptive metacognition and their relationships with emotional distresses. The results showed that DMN might serve as the general network associated with CSC, NC, and NEG; nevertheless, the distinct connection patterns suggested that each subdimension relies on some specific nodes or FC edges. We also proved the mediating role of CSC, NC and NEG subdimension in the relationship between its FC basis and emotional distresses. Further, the DMN–CON/VIS FCs of CSC could be effective in predicting the increase of individual anxiety, the DMN–VAN/VIS FCs of NEG could predict the longitudinal change of PA, while the FCs within DMN or between VIS–SMH were significantly associated with the change of NA.

### 4.1. Common and Distinct Neural Correlates of Three Metacognition Subdimensions in MCQ-30

It is well documented that DMN is crucial in self-referential processing and higher cognitive functions [68, 69] and its key nodes such as medial PFC [17, 70, 71], precuneus, and posterior cingulate cortex [18, 72] were highly related to individual metacognition. Our results here indicated that the FC networks of the metacognition subdimensions were mainly involved in the DMN, which is consistent with a previous study [25]. These evidences mirrored that aberrant FC patterns of DMN could be closely linked with dysfunction of self-integration and negative thought patterns, which further lead to a series of emotional distresses [6, 7] such as depression [73, 74] and anxiety disorders [75].

Nevertheless, as the five subdimensions show different aspects of metacognition, their distinct FC patterns may be unsurprising. In accordance with Garcia-Cordero et al. [37], the FC network of CSC dimension relied on the correlates between the higher systems (DMN, CON, and DAN) or their large-scale connectivity with the primary systems (AUD and VIS), suggesting that FCs between the fronto-posterior network were associated with metacognitive monitoring abilities. Moreover, our findings might indicate that the cognitive self-awareness related to the precentral gyrus is the crucial part of CSC. The precentral gyrus is responsible for extracting information related to individual cognitive experience, and regulating their thoughts and behavior [76, 77]. Abnormality of the right precentral gyrus was consistently connected with negative self-thoughts in depression patients [78].

Besides, the majority of the FC network of NC dimension was between DMN–VAN, which partly aligns with the finding of Lu et al. [79]. As it illustrated, thought suppression was closely related to the structure of the right superior frontal gyrus and the FC between the right superior frontal gyrus and the left middle temporal gyrus. These two are, respectively, the key regions of the DMN and the VAN [8]. Together with VIS, VAN is responsible for switching attention, evaluating visual information, and responding to sudden signals [80–82]. This network is believed to be related to the cognitive need for control, and further serves as a potential risk factor for emotional distresses. Hence, the significant contribution of FC intra DMN, VIS, and inter DMN–VAN to the NC dimension could be possible in this study.

NEG dimension is one of the core features of anxiety symptoms [5, 83] and is often accompanied by aberrant FC within DMN [84, 85] and VIS/visual cortex [86] and FC between these two networks [87]. Our finding that the FC network of NEG is mainly between DMN–VIS provides further evidence. Additionally, the middle temporal gyrus is known as the key to sensory-related mental representations and cognitive processing of emotions [88] and is linked to worry-dominated anxiety symptoms [89, 90]. The right superior temporal gyrus is the key node of both NEG and NC dimensions, which reflects its importance to maladaptive metacognition [35] and negative emotional experience [91]. It can be seen that the NEG dimension relates to maladaptive thought patterns, highlighting its prominent role in the maintenance and development of various emotional distresses.

Compared to our main findings based on the Power 264 atlas, there are indeed some differences among the results derived from different parcellations, which are likely due to inherent differences in regional segmentation approaches. Such variations suggested that the neural substrates of metacognition subdimensions may demonstrate differential sensitivity to particular parcellation. Notably, despite these variations, the consistent identification of FC networks associated with NC and NEG subdimensions across all three parcellation schemes supported the robustness of these FC networks as reliable neural indicators of metacognition.

### 4.2. Mediating Effect of CSC, NC, and NEG

Compared with the other two subdimensions, neural connectivity bases of CSC, NC, and NEG are indirectly linked with anxiety and NA through the emergence and development of maladaptive metacognition. Excessive worry is regarded as one of the core facets of anxiety, underlying negative metacognitive beliefs about danger and perceived incompetence might make individuals more vulnerable to anxiety disorder [4]. Specifically, altered CSC FC is likely to induce dysregulation of self-related cognition and emotion, resulting in an abnormally strong self-focus and excessive worry [35], while NC involves the control and inhibition of negative thinking. If negative self-relevant information is always prioritized and becomes perseverative, the networks responsible for signal sensing, attention maintenance, information integration, and motion adjustment will be overloaded, eventually leading to obvious anxiety-related symptoms.

Among three subdimensions, the mediating effect of NEG is salient in the relationships between its FC network and all four affective outcomes. Namely, as the consequences of aberrant connectivity between DMN and sensor cortex systems, negative metacognitive beliefs of uncontrollability and danger further lead to both anxiety and depression across physical illnesses [11, 12, 92]. Our finding suggested that beliefs concerning the controllability of cognition are considered central to self-regulation [93]. It also provides empirical evidence to the notes that DMN is the center for integrating external and internal information, other than an “intrinsic” system in daily life [21]. Thus, the dysconnectivity of DMN and some other networks underlying action, physiological control, arousal, and sensory signaling is widespread and fundamental, hypothesized to play a role across most types of psychological disorders.

### 4.3. The Functional Neural Basis of CSC and NEG Subdimension Predict Longitudinal Changes in Emotional Distresses

The predictive longitudinal effects of CSC and NEG bolster our perspective that negative metacognition beliefs regarding the uncontrollability of thoughts, along with the monitoring of metacognitive processes, could hold significant importance to psychological dysfunction. Psychological disorders are strongly linked with a reduction in dynamic cognitive control and adaptability, which may be related to the reconfiguration of connectivity patterns of attention and control networks [80]. Individuals who need to implement continuous top–down preparatory control often have uncertainty about themselves, a sense of uncontrollability and excessive worry about the future. Altered sensitive cognition pattern has the potential to not only cause immediate emotional maladjustment but also contribute to persistent emotional distresses in the long run.

Based on the perspective of the meta-cognitive model of psychological disorders, the robust and reliable associations found for metacognition (particularly the CSC and NEG), may imply that while treating the emotional distress issues of non-clinical, regulating negative metacognition beliefs about their self-relevant thoughts and awareness might be the key point [6]. Metacognitive therapy potentially enhances self-awareness, enabling a precise identification and in-depth comprehension of personal anxieties and their underlying cognitive frameworks, further promoting the acceptance and healthy processing of their concerns. Ultimately, it contributes to remodeling maladaptive thought patterns and modifying metacognition [93], making individuals cope with daily challenges and stresses in a healthier and more positive way.

In addition, our longitudinal data revealed differential patterns of emotional changes from baseline to follow-up measurement. That would be the reason that the participants in our study were all freshmen and sophomores from a top-tier university in China. The increase in depressive symptoms may be particularly pronounced among high-achieving university students. After the initial transition period, these students often face a significant gap between their high expectations and the demanding realities of elite academic environments [94]. Meanwhile the competitive atmosphere may foster intensive peer comparison and self-evaluation, potentially leading to increased self-doubt and academic-reality discrepancies, both of which are well-documented risk factors for depression [95, 96]. The external stressors experienced may also activate maladaptive metacognitive patterns, such as worry and rumination. Such CAS central components would significantly contribute to the development and persistence of emotional distress (e.g., depression) [97].

Conversely, the observed decrease in anxiety might be attributed to the adaptive capabilities of those high-achieving students, who often possess strong executive functioning skills, such as planning, self-efficacy, and perceived control over future outcomes. These cognitive resources may act as protective factors against anxiety as students progress through their academic years [98].

Our findings provide novel insights into how intrinsic brain connectivity networks may serve as a mechanistic framework for understanding metacognitive processes and emotional regulation. Specifically, the identification of DMN as a common neural substrate across three metacognitive subdimensions suggests its fundamental role in self-referential processing and introspective monitoring. The distinct connectivity patterns observed for each subdimension—particularly the CSC-related frontoparietal network interactions, NC-related DMN–VAN coupling, and NEG-related DMN–visual network integration—reveal how different aspects of metacognitive processing may emerge from specific network-level organizations. This network-based approach has been successfully applied to understand various cognitive and clinical phenomena, from decision impulsivity [31] to complex social cognitive dysfunction in psychiatric disorders [27].

Moreover, these network-behavior relationships were further validated by their mediating effects between FC and emotional distresses, as well as their predictive power for anxiety and affect changes. This network-based framework helps bridge the gap between brain organization and behavioral manifestations by demonstrating how distributed neural systems working in concert, rather than isolated regions, give rise to complex cognitive processes. Recent studies have demonstrated the utility of this approach in characterizing both state and trait characteristics of psychiatric symptoms [30]. The excessive FC patterns we observed, especially in CSC and NEG-related networks, may represent a neural mechanism by which altered network interactions lead to impaired self-monitoring and increased vulnerability to affective disorders. These findings align with the growing recognition that psychiatric symptoms often reflect disruptions in coordinated brain network function rather than focal brain abnormalities, providing potential targets for network-based interventions.

There are some limitations to be noted. First, the sample size may not be sufficiently large, and the study is limited to undergraduates from a specific region, which may restrict the generalizability of our findings to a broader population. The sensitivity analysis results for feature selection *p*-value thresholds suggest that 0.01 may be a good threshold, but external validation is needed. Additionally, external datasets could be utilized in future research to enhance the reliability and generalizability of the results. Second, only one positive mental health variable (PA) was considered, which limits the ability to fully understand the relationship between metacognition and positive emotional states. Furthermore, although the main findings of this study were largely validated through tenfold cross-validation, some inconsistencies remain. Thus, further validation with larger and more diverse samples is necessary to confirm the robustness of these results. Brain structure information was not focused on in the current study. To better explore the neural basis of metacognition, future multimodal studies combining brain structure data need to be conducted. Moreover, future research could explore the integration of independent components analysis (ICA)–based networks or dynamic FC within the CPM framework [99–101], offering valuable insights into brain organization and its temporal dynamics. These approaches may further illuminate the complex neural mechanisms underlying metacognition.

## 5. Conclusion

In general, this study mainly explores the neural basis of metacognition subdimensions and their associations with emotional distresses. Our findings demonstrated the common and distinct FC bases of maladaptive metacognition. The abnormal FCs of CSC and NEG might be responsible for impaired self-check-related ability and further increase the risk of several emotional distresses.

## Figures and Tables

**Table 1:** Results of correlation analyses among subdimensions of MCQ-30, PANAS, BAI, and BDI in t1.

Measures	1	2	3	4	5	6	7	8	9
1 MCQ-30	1	—	—	—	—	—	—	—	—
2 CC	0.613*⁣*^*∗∗∗*^	1	—	—	—	—	—	—	—
3 POS	0.446*⁣*^*∗∗∗*^	0.122	1	—	—	—	—	—	—
4 CSC	0.661*⁣*^*∗∗∗*^	0.168*⁣*^*∗*^	0.246*⁣*^*∗∗*^	1	—	—	—	—	—
5 NEG	0.684*⁣*^*∗∗∗*^	0.286*⁣*^*∗∗∗*^	−0.014	0.296*⁣*^*∗∗∗*^	1	—	—	—	—
6 NC	0.684*⁣*^*∗∗∗*^	0.292*⁣*^*∗∗∗*^	0.013	0.293*⁣*^*∗∗∗*^	0.567*⁣*^*∗∗∗*^	1	—	—	—
7 PA	0.035	−0.035	0.188*⁣*^*∗*^	0.137	−0.151*⁣*^*∗*^	−0.042	1	—	—
8 NA	0.417*⁣*^*∗∗∗*^	0.317*⁣*^*∗∗∗*^	−0.014	0.168*⁣*^*∗*^	0.450*⁣*^*∗∗∗*^	0.376*⁣*^*∗∗∗*^	−0.169*⁣*^*∗*^	1	—
9 BAI	0.406*⁣*^*∗∗∗*^	0.282*⁣*^*∗∗∗*^	0.041	0.184*⁣*^*∗*^	0.368*⁣*^*∗∗∗*^	0.388*⁣*^*∗∗∗*^	−0.197*⁣*^*∗∗*^	0.504*⁣*^*∗∗∗*^	1
10 BDI	0.304*⁣*^*∗∗∗*^	0.203*⁣*^*∗∗*^	−0.169*⁣*^*∗*^	0.070	0.467*⁣*^*∗∗∗*^	0.390*⁣*^*∗∗∗*^	−0.443*⁣*^*∗∗∗*^	0.480*⁣*^*∗∗∗*^	0.528*⁣*^*∗∗∗*^

*Note*: Age, gender, and head motion were controlled as the covariates. *⁣*^*∗*^*p* < 0.05, *⁣*^*∗∗*^*p* < 0.01, *⁣*^*∗∗∗*^*p* < 0.001, two-tailed. MCQ-30, Metacognition Questionnaire-30 total score; NA, negative affect of PANAS (positive and negative affect schedule); NC, need to control thoughts; NEG, negative beliefs about uncontrollability and danger of worry; PA, positive affect of PANAS; POS, positive beliefs about worry.

Abbreviations: BAI, beck anxiety inventory; BDI, beck depression inventory; CC, cognitive confidence; CSC, cognitive self-consciousness.

**Table 2:** Predictive performance of positive-correlated FC networks for the MCQ-30 total score and its five subdimensions with false discovery rate (FDR) correction.

Predictive performance	MCQ-30	CC	POS	CSC	NEG	NC
Positive-correlated FC network						
* r*	−0.042	0.021	−0.160	0.204*⁣*^*∗*^	0.015	0.238*⁣*^*∗*^
* p*_perm_	0.621	0.459	0.877	**0.033**	0.462	**0.015**
FDR corrected *p* value	—	0.231	0.351	**0.033**	0.231	**0.030**
Number of selected edges	177	155	101	194	218	271
Negative-correlated FC network						
* r*	0.138	0.032	−0.137	−0.011	0.212*⁣*^*∗*^	0.135
* p*_perm_	0.128	0.408	0.859	0.530	**0.023**	0.144
FDR corrected *p* value	—	0.530	0.687	0.530	0.092	0.288
Number of selected edges	172	187	96	156	254	258

*Note*: The predictive performance was evaluated by Pearson's correlation *r* between the real score and the predicted score derived from the CPM model. *p*_perm_ denotes the *p*-value resulting from a permutation test with 1000 repetitions. Model prediction performance with *p* < 0.05 is marked in bold. Number of selected edges represents the number of significant edges selected by the CPM model. *⁣*^*∗*^*p* < 0.05, two-tailed. MCQ-30, Metacognition Questionnaire-30 total score; NC, need to control thoughts; NEG, negative beliefs about uncontrollability and danger of worry; POS, positive beliefs about worry.

Abbreviations: CC, cognitive confidence; CSC, cognitive self-consciousness.

**Table 3:** Results of mediation analyses with CSC, NC, and NEG as mediator.

Mediation pathways	Total effect	Direct effect	Indirect effect	95% CI of indirect effect
CSC				
FC-CSC-BAI	0.083	−0.083	0.166	**[0.066, 0.311]**
FC-CSC-BDI	0.021	−0.052	0.073	[−0.055, 0.203]
FC-CSC-PA	0.182	0.163	0.019	[−0.115, 0.152]
FC-CSC-NA	0.040	−0.142	0.182	**[0.044, 0.313]**
NC				
FC-NC-BAI	0.228	−0.138	0.366	**[0.232, 0.497]**
FC-NC-BDI	0.251	−0.086	0.337	**[0.186, 0.489]**
FC-NC-PA	−0.026	0.011	–0.037	[−0.206, 0.134]
FC-NC-NA	0.183	−0.217	0.399	**[0.255, 0.545]**
NEG				
FC-NEG-BAI	−0.176	0.139	−0.315	**[−0.433, -0.201]**
FC-NEG-BDI	−0.266	0.100	−0.366	**[−0.501, −0.235]**
FC-NEG-PA	0.008	−0.176	0.184	**[0.034, 0.340]**
FC-NEG-NA	−0.219	−0.096	−0.380	**[−0.508, −0.257]**

*Note*: The value of the independent variable (i.e., FC) is the mean value of the FC edges that can predict the score of the specific metacognition subdimension in the prediction model. The mediating effect is valid, if the bootstrapped (5000 times) 95% confidence interval of the coefficient does not contain 0. The bold letter represents a significant mediation effect; the coefficients above are all standardized. NA, negative affect of PANAS (positive and negative affect schedule); NC, need to control thoughts; NEG, negative beliefs about uncontrollability and danger of worry; PA, positive affect of PANAS.

Abbreviations: BAI, beck anxiety inventory; BDI, beck depression inventory; CSC, cognitive self-consciousness.

## Data Availability

The data that support the findings of this study are available upon request from the corresponding author.

## Supporting Information

Supporting Information Connectome-based predictive modeling longitudinal prediction analysis. Table S1. Difference test for longitudinal changes of retest data. Table S2. Node degree of CSC positive-correlated FC networks. Table S3. Node degree of NC positive-correlated FC networks. Table S4. Node degree of NEG negative-correlated FC networks. Table S5. Node and network information of overlapped edges between the CSC and NC FC networks. Table S6. Results of mediation analyses with t2 emotional outcomes (PANAS, BAI, and BDI scores) as dependent variables. Table S7. Results of prediction analyses with FC networks of the MCQ-30 total score and its five subdimensions basing on 5000 permutation tests. Table S8. Results of prediction analyses with FC network of the MCQ-30 total score and its five subdimensions using tenfold cross-validation. Table S9. Results of prediction analyses with FC network after scrubbing operation of the MCQ-30 total score and its five subdimensions. Table S10. Results of prediction analyses with FC network of CSC subdimension under different *p* thresholds for edge selection. Table S11. Results of prediction analyses with FC network of NC subdimension under different *p* thresholds for edge selection. Table S12. Results of prediction analyses with FC network of NEG subdimension under different *p* thresholds for edge selection. Table S13. Results of prediction analyses with FC networks of the MCQ-30 total score and its five subdimensions using 268-node atlas. Table S14. Results of prediction analyses with FC networks of the MCQ-30 total score and its five subdimensions using the 246-node atlas.
