# Supplementary material for: The Specificity of Metacognition Questionnaire-30 Subdimensions: Findings From Connectome-Based Predictive Modeling
Source: Depress Anxiety. 2025 Aug 19;2025:5581270. doi: 10.1155/da/5581270 (PMC12381410; doi:10.1155/da/5581270)
Supplement: Supporting Information — Connectome-based predictive modeling longitudinal prediction analysis. Table S1. Difference test for longitudinal changes of retest data. Table S2. Node degree of CSC positive-correlated FC networks. Table S3. Node degree of NC positive-correlated FC networks. Table S4. Node degree of NEG negative-correlated FC networks. Table S5. Node and network information of overlapped edges between the CSC and NC FC networks. Table S6. Results of mediation analyses with t2 emotional outcomes (PANAS, BAI, and BDI scores) as dependent variables. Table S7. Results of prediction analyses with FC networks of the MCQ-30 total score and its five subdimensions basing on 5000 permutation tests. Table S8. Results of prediction analyses with FC network of the MCQ-30 total score and its five subdimensions using tenfold cross-validation. Table S9. Results of prediction analyses with FC network after scrubbing operation of the MCQ-30 total score and its five subdimensions. Table S10. Results of prediction analyses with FC network of CSC subdimension under different p thresholds for edge selection. Table S11. Results of prediction analyses with FC network of NC subdimension under different p thresholds for edge selection. Table S12. Results of prediction analyses with FC network of NEG subdimension under different p thresholds for edge selection. Table S13. Results of prediction analyses with FC networks of the MCQ-30 total score and its five subdimensions using 268-node atlas. Table S14. Results of prediction analyses with FC networks of the MCQ-30 total score and its five subdimensions using the 246-node atlas. [file 5581270.f1.docx]

**The Specificity of Metacognition Questionnaire-30 Subdimensions: Findings from Connectome-Based Predictive Modeling**

Ruocen Hu^1#^, Meng Yu^2#^, Liangfang Li^1#^, Hui He^1^, Sihan Wei^1^, Junji Ma^1^, Yue Gu^1^, Zhengjia Dai^1*^

**Supplementary materials**

**Supplementary methods:**

Connectome-Based Predictive Modeling

Longitudinal prediction analysis

**Supplementary results:**

**Table S1** Difference test for longitudinal changes of retest data.

**Table S2** Node degree of CSC positive-correlated FC networks.

**Table S3** Node degree of NC positive-correlated FC networks.

**Table S4** Node degree of NEG negative-correlated FC networks.

**Table S5** Node and network information of overlapped edges between the CSC and NC FC networks.

**Table S6** Results of mediation analyses with t2 emotional outcomes (PANAS, BAI, and BDI scores) as dependent variables.

**Table S7** Results of prediction analyses with FC networks of the MCQ-30 total score and its five subdimensions basing on 5000 permutation tests.

**Table S8** Results of prediction analyses with FC network of the MCQ-30 total score and its five subdimensions using 10-fold cross-validation.

**Table S9** Results of prediction analyses with FC network after scrubbing operation of the MCQ-30 total score and its five subdimensions.

**Table S10** Results of prediction analyses with FC network of CSC subdimension under different *p* thresholds for edge selection.

**Table S11** Results of prediction analyses with FC network of NC subdimension under different *p* thresholds for edge selection.

**Table S12** Results of prediction analyses with FC network of NEG subdimension under different *p* thresholds for edge selection.

**Table S13** Results of prediction analyses with FC networks of MCQ-30 total score and its five subdimensions using 268-node atlas.

**Table S14** Results of prediction analyses with FC networks of MCQ-30 total score and its five subdimensions using 246-node atlas.

## Supplementary methods

**2.1** **Connectome-Based Predictive Modeling**

Specifically, the major process steps of CPM include: (1) Feature selection. First, the data of each subject is taken as the testing set in turn, and the data of the other subjects is taken as the training set. Then the FC edge that was significantly correlated (gender, age, and head motion were covariables) with the score of metacognition subdimension was selected as the feature edge (threshold = 0.01). Both positive and negative correlated edges were included. (2) Feature summary. Calculate the sum of the strength of all feature edges. (3) Model construction. Two linear regression prediction models were established for the feature edges (positive and negative correlated edges), the edge strength is taken as the independent variable and the metacognition subdimension score is taken as the dependent variable. In each iteration, *r* and *p* values of Pearson correlation analysis between the predicted and real values of the metacognition scores are calculated, and significant positive *r* values are used as valid results. Thus, the corresponding FCs that can significantly predict metacognition are selected. (4) Prediction significance evaluation. Finally, 1000 times permutation tests are used to evaluate prediction significance to determine whether the results of the prediction model are significantly better than those generated randomly.

**2.2 Longitudinal prediction analysis**

The specific prediction steps are as follows: (1) The FC network of the CSC, NEG and NC are made into three masks (264 × 264 binary matrix); (2) Multiply these three masks by the FC matrix; (3) Then, FC edges that are significantly correlated with mental health change scores are selected as feature edges with a same threshold of 0.01, and the sum of strength of all feature edges are calculated; (4) Then model fitting was carried out through leave-one cross-validation. As in this step, we directly use FC network of the CSC, NEG, and NC subdimension (CSC: 194 edges; NEG: 254 edges; NC: 271 edges) instead of the original FC matrix (34,848 edges) with a threshold of 0.01, resulting in a small number of feature edges. Therefore, we adopted a regression model that could combine positive and negative correlated edges for fitting in this step to focus on more feature edges at the same time.

## Supplementary results

**3.1 Description and mental health assessment**

The paired samples *t*-test of repeated mental health assessments showed the longitudinal changes in mental health scores. Specifically, the scores of PA and BAI were significantly decreased (*p* = 0.027 and *p* < 0.001, respectively) while BDI was increased (*p* < 0.001).

**Table S1** Difference test for longitudinal changes of retest data.

|  | Initial test (T1) | | Retest (T2) | | Paired sample *t*-test | |
| --- | --- | --- | --- | --- | --- | --- |
|  | α | *M* ± *SD* | α | *M* ± *SD* | *t*_(158)_ | *p* |
| MCQ-30 | 0.860 | 64.03 ± 11.25 | 0.874 | 63.96 ± 11.58 | 0.323 | 0.747 |
| CC | 0.692 | 12.55 ± 3.82 | 0.817 | 12.44 ± 3.79 | 0.527 | 0.599 |
| POS | 0.783 | 12.47 ± 3.55 | 0.867 | 12.31 ± 3.41 | 0.955 | 0.341 |
| CSC | 0.846 | 14.58 ± 3.89 | 0.868 | 14.29 ± 3.92 | 0.704 | 0.482 |
| NEG | 0.875 | 12.53 ± 3.54 | 0.857 | 12.88 ± 3.80 | ­1.466 | 0.145 |
| NC | 0.828 | 11.83 ± 3.34 | 0.771 | 12.05 ± 3.61 | ­0.908 | 0.365 |
| PA | 0.891 | 25.67 ± 5.84 | 0.885 | 24.72 ± 5.70 | 2.231 | **0.027** |
| NA | 0.763 | 19.38 ± 5.11 | 0.863 | 19.64 ± 7.04 | 0.241 | 0.810 |
| BAI | 0.847 | 11.47 ± 7.24 | 0.900 | 8.91 ± 7.42 | 3.675 | **＜0.001** |
| BDI | 0.862 | 7.41 ± 5.64 | 0.896 | 12.18 ± 8.36 | ­7.015 | **＜0.001** |

*Note*. α, Cronbach's α coefficients; M, mean value; SD, standard deviation. MCQ-30, Metacognition Questionnaire-30 total score; CC, Cognitive Confidence; POS: Positive Beliefs about Worry; CSC, Cognitive Self-Consciousness; NEG, Negative Beliefs about Uncontrollability and Danger of Worry; NC, Need to Control Thoughts. PA, positive affect of Positive and Negative Affect Schedule; NA, negative affect of Positive and Negative Affect Schedule; BAI, Beck Anxiety Inventory; BDI, Beck Depression Inventory.

**3.2 FC network of metacognition subdimensions**

**Table S2** Node degree of CSC positive-correlated FC networks.

| No. | ROI | Degree | Node | MNI coordinate | | | Lobe | Network |
| --- | --- | --- | --- | --- | --- | --- | --- | --- |
|  |  |  |  | x | y | z |  |  |
| 1 | 264 | 11 | Precentral_R  (BA6) | 29 | -5 | 54 | R-MotorStrip | Dorsal attention |
| 2 | 195 | 11 | Angular_L  (BA39) | -42 | -55 | 45 | L-Parietal | Fronto-parietal Task Control |
| 3 | 185 | 11 | Cerebelum_Crus1_R | 35 | -67 | -34 | Cerebellum | Uncertain |
| 4 | 99 | 9 | Frontal_Sup_L  (BA8) | -16 | 29 | 53 | L-Prefrontal | Default Mode |
| 5 | 74 | 9 | Occipital_Mid_L  (BA19) | -41 | -75 | 26 | L-Occipital | Default Mode |
| 6 | 49 | 8 | Frontal_Sup_R  (BA6) | 19 | -8 | 64 | R-MotorStrip | Cingulo-opercular Task Control |
| 7 | 86 | 8 | Angular_L  (BA39) | -44 | -65 | 35 | L-Parietal | Default Mode |
| 8 | 243 | 7 | Cerebelum_6_R | -16 | -65 | -20 | Cerebellum | Cerebellar |
| 9 | 172 | 7 | Fusiform_L | -33 | -79 | -13 | L-Occipital | Visual |
| 10 | 121 | 6 | Frontal_Sup_R  (BA8) | 13 | 30 | 59 | R-Prefrontal | Default Mode |
| 11 | 63 | 9 | Temporal_Sup_R  (BA23) | 58 | -16 | 7 | R-Temporal | Auditory |
| 12 | 184 | 10 | Cerebelum_Crus2_R | 17 | -80 | -34 | R-Cerebellum | Uncertain |

*Note*. ROI, ROI ID in Power-264 template. L, left; R, right. BA, Brodmann area; MNI, Montreal Neurological Institute.

**Table S3** Node degree of NC positive-correlated FC networks.

| No. | ROI | Degree | Node | MNI coordinate | | | Lobe | Network |
| --- | --- | --- | --- | --- | --- | --- | --- | --- |
|  |  |  |  | x | y | z |  |  |
| 1 | 238 | 38 | Temporal_Sup_R  (BA22) | 52 | -33 | 8 | R-Temporal | Ventral attention |
| 2 | 264 | 12 | Precentral_R  (BA38) | 29 | -5 | 54 | R-MotorStrip | Dorsal attention |
| 3 | 108 | 11 | Frontal_Sup_Medial_R(BA10) | 9 | 54 | 3 | R-Prefrontal | Default Mode |
| 4 | 237 | 9 | Temporal_Sup_L  (BA22) | -55 | -40 | 14 | L-Temporal | Ventral attention |
| 5 | 201 | 9 | Frontal_Inf_Tri_L(BA9) | -42 | 25 | 30 | L-Prefrontal | Fronto-parietal Task Control |
| 6 | 92 | 9 | Cingulum_Post_R(BA23) | 8 | -48 | 31 | R-Limbic | Default Mode |
| 7 | 197 | 9 | Frontal_Mid_L  (BA10) | -34 | 55 | 4 | L-Prefrontal | Fronto-parietal Task Control |
| 8 | 145 | 8 | Calcarine_R  (Primvisual17) | 8 | -72 | 11 | R-Occipital | Visual |
| 9 | 29 | 8 | Postcentral_R  (BA6) | 44 | -8 | 57 | R-Occipital | Sensory/somatomotor Hand |
| 10 | 115 | 7 | Frontal_Sup_Medial_L(BA9) | -8 | 48 | 23 | L-Prefrontal | Default Mode |
| 11 | 118 | 7 | Temporal_Mid_L(BA21) | -58 | -30 | -4 | L-Temporal | Default Mode |
| 12 | 221 | 7 | Cingulate_Post_R(BA23) | 2 | -24 | 30 | L-Limbic | Memory retrieval |

*Note*. ROI, ROI ID in Power-264 template. L, left; R, right. BA, Brodmann area; MNI, Montreal Neurological Institute.

**Table S4** Node degree of NEG negative-correlated FC networks.

| No. | ROI | Degree | Node | MNI coordinate | | | Lobe | Network |
| --- | --- | --- | --- | --- | --- | --- | --- | --- |
|  |  |  |  | x | y | z |  |  |
| 1 | 238 | 24 | Temporal_Sup_R  (BA22) | 52 | -33 | 8 | R-Temporal | Ventral attention |
| 2 | 118 | 11 | Temporal_Mid_L(BA21) | -58 | -30 | -4 | L-Temporal | Default Mode |
| 3 | 40 | 10 | Supp_Motor_Area_R(BA6) | 3 | -17 | 58 | R-MotorStrip | Sensory/somatomotor Hand |
| 4 | 257 | 8 | Temporal_Inf_R  (Fusiform37) | 46 | -59 | 4 | R-Temporal | Dorsal attention |
| 5 | 161 | 8 | Temporal_Inf_R  (BA19) | 42 | -66 | -8 | R-Occipital | Visual |
| 6 | 41 | 8 | Precentral_R  (Primmotor4) | 38 | -17 | 45 | R-MotorStrip | Sensory/somatomotor Hand |
| 7 | 247 | 8 | Fusiform_R  (Parahip36) | 33 | -12 | -34 | R-Limbic | Uncertain |
| 8 | 21 | 8 | Precentral_R  (BA21) | 29 | -17 | 71 | R-MotorStrip | Sensory/somatomotor Hand |
| 9 | 98 | 7 | Frontal_Sup_Medial_L(BA8) | -10 | 39 | 52 | L-Prefrontal | Default Mode |
| 10 | 81 | 7 | Temporal_Pole_Mid_L(BA38) | -44 | 12 | -34 | L-Temporal | Default Mode |
| 11 | 34 | 7 | Postcentral_L  (Primsensory1) | -21 | -31 | 61 | L-Parietal | Sensory/somatomotor Hand |
| 12 | 97 | 7 | Frontal_Sup_R  (BA8) | 23 | 33 | 48 | R-Prefrontal | Default Mode |

*Note*. ROI, ROI ID in Power-264 template. L, left; R, right. BA, Brodmann area; MNI, Montreal Neurological Institute.

**Table S5** Node and network information of overlapped edges between the CSC and NC FC networks.

| Node information | Network information |
| --- | --- |
| Angular_L--Frontal_Sup_R | DMN-CON |
| Angular_L--Frontal_Sup_R | FPN-CON |
| Temporal_Sup_R--Frontal_Med_Orb_R | VAN-DMN |
| Precentral_R--Angular_L | DAN-DMN |
| Precentral_R--Frontal_Sup_L | DAN-DMN |
| Frontal_Inf_Orb_R--Angular_R | FPN-FPN |

*Note*. L, left; R, right. DMN, Default Mode Network; CON, Cingulo-opercular Task Control Network; FPN, Fronto-parietal Task Control Network; VAN, Ventral Attention Network; DAN, Dorsal Attention Network.

**3.3 Mediation analyses with t2 emotional outcomes as dependent variables**

**Table S6** Results of mediation analyses with t2 emotional outcomes (PANAS, BAI, and BDI scores) as dependent variables.

|  | Total effect | Direct effect | Indirect effect | 95%CI of Indirect effect |
| --- | --- | --- | --- | --- |
| CSC | | | | |
| FC-CSC-BAI | 8.539 | 7.116 | 1.423 | [-1.114, 4.835] |
| FC-CSC-BDI | -5.400 | -4,972 | -0.428 | [-4.541, 2.357] |
| FC-CSC-PA | 18.424 | 18.887 | -0.463 | [-2.490, 1.266] |
| FC-CSC-NA | -4.589 | -5.124 | 0.535 | [-2.686, 2.895] |
| NEG | | | | |
| FC-NEG-BAI | -13.902 | -13.209 | -0.692 | [-4.393, 1.575] |
| FC-NEG-BDI | -15.234 | -14.724 | -0.511 | [-3.697, 1.629] |
| FC-NEG-PA | -10.530 | -10.725 | 0.195 | [-1.052, 2.089] |
| FC-NEG-NA | -5.407 | -4.951 | -0.456 | [-3.211, 1.295] |
| NC | | | | |
| FC-NC-BAI | 25.263 | 24.925 | 0.338 | [-1.250, 2.832] |
| FC-NC-BDI | 9.819 | 10.428 | -0.610 | [-3.822, 1.397] |
| FC-NC-PA | -2.561 | -2.675 | 0.114 | [-1.266, 1.938] |
| FC-NC-NA | 8.975 | 9.783 | -0.809 | [-3.831, 1.216] |

*Note*. The mediation analyses examined whether t1 metacognition subdimensions mediated the relationship between t1 FC patterns (using the mean value of the significant edges from our prediction model) and t2 emotional outcomes (PANAS, BAI, and BDI scores). Age, gender, and head motion were controlled as covariates. CSC, Cognitive Self-Consciousness; NEG, Negative Beliefs about Uncontrollability and Danger of Worry; NC, Need to Control Thoughts. PA, positive affect of Positive and Negative Affect Schedule; NA, negative affect of Positive and Negative Affect Schedule; BAI, Beck Anxiety Inventory; BDI, Beck Depression Inventory.

**3.4 CPM results: Predictive performance of FC networks basing on 5000 permutation tests**

**Table S7** Results of prediction analyses with FC networks of the MCQ-30 total score and its five subdimensions basing on 5000 permutation tests.

|  | MCQ-30 | CC | POS | CSC | NEG | NC |
| --- | --- | --- | --- | --- | --- | --- |
| Positive-correlated FC network | | | | | | |
| *r* | –0.042 | 0.021 | –0.160 | 0.204^*^ | 0.015 | 0.238^*^ |
| *p*_perm_ | 0.631 | 0.446 | 0.865 | **0.032** | 0.477 | **0.014** |
| FDR corrected *p* value | / | 0.238 | 0.346 | **0.032** | 0.238 | **0.028** |
| Negative-correlated FC network | | | | | | |
| *r* | 0.138 | 0.032 | –0.137 | –0.011 | 0.212^*^ | 0.135 |
| *p*_perm_ | 0.127 | 0.414 | 0.826 | 0.559 | **0.020** | 0.137 |
| FDR corrected *p* value | / | 0.552 | 0.661 | 0.559 | **0.080** | 0.274 |

*Note*. The predictive performance was evaluated by Pearson’s correlation *r* between the real score and the predicted score derived from the CPM model. Model predictive performance with *p* < 0.05 is marked in bold. MCQ-30, Metacognition Questionnaire-30; CC, Cognitive Confidence; POS, Positive Beliefs about Worry; CSC, Cognitive Self-Consciousness; NEG, Negative Beliefs about Uncontrollability and Danger of Worry; NC, Need to Control Thoughts. **p* < 0.05, two tailed.

**3.5 Validation analysis**

**Table S8** Results of prediction analyses with FC network of the MCQ-30 total score and its five subdimensions using 10-fold cross-validation.

|  | MCQ-30 | CC | POS | CSC | NEG | NC |
| --- | --- | --- | --- | --- | --- | --- |
| Positive-correlated FC network | | | | | | |
| *r* | 0.045 | -0.059 | -0.104 | 0.059 | 0.088 | 0.191* |
| *p*_perm_ | 0.332 | 0.658 | 0.797 | 0.276 | 0.201 | **0.024** |
| FDR corrected *p* value | / | 0.638 | 0.638 | 0.368 | 0.368 | 0.096 |
| Negative-correlated FC network | | | | | | |
| *r* | 0.097 | 0.029 | -0.171 | -0.001 | 0.215* | 0.091 |
| *p*_perm_ | 0.166 | 0.380 | 0.910 | 0.520 | **0.006** | 0.180 |
| FDR corrected *p* value | / | 0.508 | 0.728 | 0.520 | **0.024** | 0.360 |

*Note*. The predictive performance was evaluated by Pearson’s correlation *r* between the real score and the predicted score derived from the CPM model. Model predictive performance with *p* < 0.05 is marked in bold. MCQ-30, Metacognition Questionnaire-30; CC, Cognitive Confidence; POS, Positive Beliefs about Worry; CSC, Cognitive Self-Consciousness; NEG, Negative Beliefs about Uncontrollability and Danger of Worry; NC, Need to Control Thoughts. **p* < 0.05, two tailed.

**Table S9** Results of prediction analyses with FC network after scrubbing operation of the MCQ-30 total score and its five subdimensions.

|  | MCQ-30 | CC | POS | CSC | NEG | NC |
| --- | --- | --- | --- | --- | --- | --- |
| Positive-correlated FC network | | | | | | |
| *r* | -0.033 | 0.009 | -0.125 | 0.206* | 0.022 | 0.228* |
| *p*_perm_ | 0.611 | 0.497 | 0.802 | **0.024** | 0.463 | **0.013** |
| FDR corrected *p* value | / | 0.249 | 0.321 | **0.024** | 0.249 | **0.024** |
| Negative-correlated FC network | | | | | | |
| *r* | 0.123 | 0.029 | -0.169 | -0.018 | 0.209* | 0.140 |
| *p*_perm_ | 0.156 | 0.419 | 0.866 | 0.616 | **0.027** | 0.122 |
| FDR corrected *p* value | / | 0.559 | 0.693 | 0.616 | 0.108 | 0.233 |

*Note*. The predictive performance was evaluated by Pearson’s correlation *r* between the real score and the predicted score derived from the CPM model. Model predictive performance with *p* < 0.05 is marked in bold. No. of selected edges represents the number of significant edges selected by the CPM model. NEG, Negative Beliefs about Uncontrollability and Danger of Worry subdimension. ^*^ *p* < 0.05, two tailed.

**Table S10** Results of prediction analyses with FC network of CSC subdimension under different *p* thresholds for edge selection.

|  | *p* threshold | | | |
| --- | --- | --- | --- | --- |
|  | 0.025 | 0.01 | 0.005 | 0.001 |
| Positive-correlated FC network | | | | |
| *r* | 0.028 | 0.204* | 0.117 | -0.305 |
| *p* | 0.712 | **0.006** | 0.117 | **<**0.001 |
| No. of selected edges | 486 | 194 | 103 | 15 |
| Negative-correlated FC network | | | | |
| *r* | 0.089 | -0.011 | 0.036 | 0.204* |
| *p* | 0.236 | 0.888 | 0.628 | **0.006** |
| No. of selected edges | 443 | 156 | 75 | 14 |

*Note*. The predictive performance was evaluated by Pearson’s correlation *r* between the real score and the predicted score derived from the CPM model. Model predictive performance with *p* < 0.05 is marked in bold. No. of selected edges represents the number of significant edges selected by the CPM model. CC, Cognitive Confidence subdimension. ^*^ *p* < 0.05, two tailed.

**Table S11** Results of prediction analyses with FC network of NC subdimension under different *p* thresholds for edge selection.

|  | *p* threshold | | | |
| --- | --- | --- | --- | --- |
|  | 0.025 | 0.01 | 0.005 | 0.001 |
| Positive-correlated FC network | | | | |
| *r* | 0.132 | 0.238* | 0.303* | 0.113 |
| *p* | 0.077 | **0.001** | **<0.001** | 0.130 |
| No. of selected edges | 612 | 271 | 163 | 40 |
| Negative-correlated FC network | | | | |
| *r* | 0.171* | 0.135 | 0.078 | -0.006 |
| *p* | **0.022** | 0.072 | 0.299 | 0.936 |
| No. of selected edges | 566 | 258 | 133 | 27 |

*Note*. The predictive performance was evaluated by Pearson’s correlation *r* between the real score and the predicted score derived from the CPM model. Model predictive performance with *p* < 0.05 is marked in bold. No. of selected edges represents the number of significant edges selected by the CPM model. NC, Need to Control Thoughts subdimension. ^*^ *p* < 0.05, two tailed.

**Table S12** Results of prediction analyses with FC network of NEG subdimension under different *p* thresholds for edge selection.

|  | *p* threshold | | | |
| --- | --- | --- | --- | --- |
|  | 0.025 | 0.01 | 0.005 | 0.001 |
| Positive-correlated FC network | | | | |
| *r* | 0.122 | 0.015 | 0.040 | -0.048 |
| *p* | 0.103 | 0.846 | 0.601 | 0.523 |
| No. of selected edges | 523 | 218 | 97 | 23 |
| Negative-correlated FC network | | | | |
| *r* | 0.191* | 0.212* | 0.121 | 0.246* |
| *p* | **0.010** | **0.004** | 0.107 | **<0.001** |
| No. of selected edges | 543 | 254 | 138 | 37 |

*Note*. The predictive performance was evaluated by Pearson’s correlation *r* between the real score and the predicted score derived from the CPM model. Model predictive performance with *p* < 0.05 is marked in bold. No. of selected edges represents the number of significant edges selected by the CPM model. NEG, Negative Beliefs about Uncontrollability and Danger of Worry subdimension. ^*^ *p* < 0.05, two tailed.

**Table S13** Results of prediction analyses with FC networks of MCQ-30 total score and its five subdimensions using 268-node atlas.

|  | MCQ-30 | CC | POS | CSC | NEG | NC |
| --- | --- | --- | --- | --- | --- | --- |
| Positive-correlated FC network | | | | | | |
| *r* | -0.100 | -0.014 | -0.034 | 0.092 | 0.051 | 0.302* |
| *p* | 0.180 | 0.850 | 0.649 | 0.219 | 0.499 | **<0.001** |
| No. of selected edges | 147 | 117 | 101 | 153 | 193 | 341 |
| Negative-correlated FC network | | | | | | |
| *r* | 0.064 | 0.080 | -0.147 | -0.027 | 0.079 | 0.193* |
| *p* | 0.393 | 0.284 | 0.049 | 0.722 | 0.288 | **0.009** |
| No. of selected edges | 161 | 209 | 124 | 156 | 223 | 275 |

*Note*. The predictive performance was evaluated by Pearson’s correlation *r* between the real score and the predicted score derived from the CPM model. Model predictive performance with *p* < 0.05 is marked in bold. MCQ-30, Metacognition Questionnaire-30; CC, Cognitive Confidence; POS, Positive Beliefs about Worry; CSC, Cognitive Self-Consciousness; NEG, Negative Beliefs about Uncontrollability and Danger of Worry; NC, Need to Control Thoughts. **p* < 0.05, two tailed.

**Table S14** Results of prediction analyses with FC networks of MCQ-30 total score and its five subdimensions using 246-node atlas.

|  | MCQ-30 | CC | POS | CSC | NEG | NC |
| --- | --- | --- | --- | --- | --- | --- |
| Positive-correlated FC network | | | | | | |
| *r* | -0.027 | -0.107 | <-0.001 | -0.008 | 0.059 | 0.296* |
| *p* | 0.720 | 0.152 | 0.992 | 0.915 | 0.429 | **<0.001** |
| No. of selected edges | 159 | 92 | 88 | 132 | 168 | 335 |
| Negative-correlated FC network | | | | | | |
| *r* | -0.005 | 0.015 | -0.096 | 0.005 | 0.165* | 0.230* |
| *p* | 0.946 | 0.838 | 0.197 | 0.947 | **0.027** | **0.002** |
| No. of selected edges | 142 | 165 | 82 | 91 | 191 | 262 |

*Note*. The predictive performance was evaluated by Pearson’s correlation *r* between the real score and the predicted score derived from the CPM model. Model predictive performance with *p* < 0.05 is marked in bold. MCQ-30, Metacognition Questionnaire-30; CC, Cognitive Confidence; POS, Positive Beliefs about Worry; CSC, Cognitive Self-Consciousness; NEG, Negative Beliefs about Uncontrollability and Danger of Worry; NC, Need to Control Thoughts. **p* < 0.05, two tailed.
